# Towards HDV Elimination Through HBV Vaccination: Global Strategies, Challenges, and Policy Gaps

**DOI:** 10.3390/vaccines14020179

**Published:** 2026-02-14

**Authors:** Enkhtuul Batbold, Naranjargal Dashdorj, Fabien Zoulim, Birke Bartosch

**Affiliations:** 1 UMR PaThLiv 1350, Inserm, Université Claude-Bernard Lyon 1, F-69003 Lyon, France; fabien.zoulim@inserm.fr; 2 The Lyon Hepatology Institute, IHU EVEREST, F-69004 Lyon, France; 3 Liver Center, Ulaanbaatar 14230, Mongolia; dashdorj@onomfoundation.org

**Keywords:** hepatitis D virus, hepatitis B virus, hepatitis B virus vaccination, viral hepatitis elimination, vaccine policy

## Abstract

Persistent infection with hepatitis D virus (HDV), also known as hepatitis delta, is considered the most severe form of chronic viral hepatitis. HDV is a defective RNA virus that depends on hepatitis B virus (HBV) for propagation. Despite its global distribution, HDV stays a neglected part of the viral hepatitis agenda, often overlooked in surveillance systems and public health policy. This oversight is particularly concerning given HDV’s aggressive clinical course, characterized by more rapid progression to cirrhosis, liver failure, and hepatocellular carcinoma (HCC) compared to HBV mono-infection. Mongolia has the highest incidence and mortality rates of HCC worldwide, with approximately 47% of cases estimated to be attributable to chronic HDV infection. Globally, an estimated 12–25 million people are co-infected with HBV and HDV, although the true prevalence is higher due to insufficient screening and incomplete data collection. Because HDV infection is entirely dependent on HBV, prevention of HBV infection through effective vaccination stands for an indirect yet highly effective strategy to curb HDV transmission. The World Health Organization (WHO), together with the global health community, has established ambitious targets to eliminate viral hepatitis as a public health threat by 2030. However, achieving HDV elimination remains particularly challenging due to limited diagnostic capacity, low awareness, and minimal inclusion of HDV in national hepatitis programs. This review explores the intersection of HDV and HBV, focusing on how expanded and optimized HBV vaccination coverage can serve as a cornerstone of global HDV prevention efforts. We examine epidemiological evidence, scientific rationale, policy developments, and key implementation challenges, with particular attention to high-burden settings such as Mongolia. Finally, we propose strategic recommendations to bridge policy and practice gaps in HDV elimination.

## 1. Background

### 1.1. Virology and Life Cycle of Hepatitis Delta Virus

Hepatitis delta virus (HDV) is a unique defective virus whose replication and transmission strictly depend on hepatitis B virus (HBV). It consists of a small, circular single-stranded negative-sense RNA genome (~1.7 kb), which encodes a single protein, the hepatitis delta antigen (HDAg). HDV relies on HBV surface antigens (HBsAg) for virion assembly, release, and entry into hepatocytes [1]. The HDV life cycle begins with HBsAg-mediated entry into hepatocytes via the sodium taurocholate cotransporting polypeptide (NTCP) receptor [2]. Once inside the cell, HDV RNA is transported to the nucleus, where it undergoes rolling-circle replication using host RNA polymerase II [3].This replication process generates both genomic and antigenomic RNA intermediates, which are processed by host enzymes, including ribozymes encoded within the HDV genome itself [4]. The hepatitis delta antigen exists in two forms, small (S-HDAg) and large (L-HDAg), which play distinct roles in regulating viral replication and virion assembly, respectively [5]. The small hepatitis delta antigen (sHDAg) promotes viral RNA replication in the nucleus, while the large hepatitis delta antigen (L-HDAg) inhibits replication and, after prenylation, mediates virion assembly by interacting with HBsAg [6,7]. Newly synthesized HDV genomes are packaged with HDAg and HBsAg to form infectious virions, which are then released from the hepatocyte to infect neighboring cells [8].

### 1.2. Natural History and Clinical Course of HDV Infection

Acute HBV/HDV co-infection typically leads to clearance of both viruses in approximately 95% of cases [9]. In contrast, HDV superinfection in individuals already infected with HBV leads to chronic HBV/HDV infection in over 90% of cases [10]. HDV is known to accelerate disease progression and increase hepatocellular carcinoma (HCC) incidence by up to 3-fold in comparison to HBV monoinfection [11,12]. At the time of diagnosis, roughly 30% to 70% of individuals with chronic hepatitis D (CHD) have cirrhosis, and over half succumb to liver-related complications within 10 years of diagnosis [13]. While global viral hepatitis causes ~1.3 million deaths annually, the proportion specifically due to HDV co-infection is not well quantified [14]. It is due to the fact that HDV is not routinely screened even among HBsAg-positive individuals in many countries, leading to both under-diagnosis and underestimation of disease burden [9,10].

### 1.3. Epidemiology and Global Burden of HDV

Worldwide, over 15 million people are estimated to be infected with HDV [15]. However, recent estimates differ widely, ranging from 12 to 40 and even up to 70 million with high prevalence in regions such as Central Asia, Africa, the Middle East, and parts of South America [16,17]. The current gap in the estimation is attributable to several factors, including limited research, low awareness, and lack of high-quality testing [18,19]. Also, differences in global HDV prevalence estimates reflect discrepancies in study method, diagnostic approaches, and population sampling. Anti-HDV antibodies indicate exposure but do not distinguish between past and current infection. In contrast, HDV RNA testing identifies active infection, typically yielding lower prevalence figures. Other factors contributing to heterogeneity include geographic biases in data collection, varieties in circulating HDV genotypes, and inconsistencies in testing sensitivity and specificity. Together, these factors underscore why reported global HDV burden ranges so widely and highlight the need for standardized, high-quality epidemiological studies.

HDV is most prevalent in regions where HBV remains endemic and public health infrastructure is limited. Central Asia, such as Mongolia, has one of the highest recorded rates of seroprevalence of HDV, with up to 60% of HBV-infected individuals testing positive for anti-HDV antibodies [20]. A study assessing the adjusted seroprevalence of hepatitis D across 25 countries found that Mongolia (61%) and Pakistan (16.6%) had a significantly higher proportion of anti-HDV positivity among HBV carriers, indicating a substantial disease burden in these regions. When adjusted for the HBV-positive population and HDV RNA positivity, China reported the highest absolute number of HDV RNA-positive cases due to its significant HBV prevalence.

In the West and Central Africa, high HDV seroprevalence is observed among HBsAg-positive populations, and varies between 6% and 69%, although national-level data are sparse [21,22]. Countries such as Moldova (18.5%) [23], Iran (2–17%) [24], and Romania (23.1%) [25] report significant HDV co-infection rates [26]. Also, the Amazon Basin and parts of Asia-Pacific showed an HDV prevalence of 41.9% amongst HBsAg carriers [27]. Together, this reveals that isolated populations with limited healthcare access exhibit localized hyperendemicity. Though less prevalent than other forms of viral hepatitis, HDV infection carries a disproportionately high burden of liver-related morbidity and mortality.

### 1.4. Current and Emerging Treatments for Chronic Hepatitis D

Treatment options for CHD remain limited. Pegylated IFN-α has modest efficacy and significant side effects, while newer approaches such as bulevirtide (BLV) [28] require prolonged therapy and are associated with frequent viral relapse after discontinuation [29,30] although the combination with Peg-IFN improves sustained responses [31]. Emerging strategies targeting HBsAg, including monoclonal antibodies, siRNA (small interfering RNA)-based therapies [32], and other entry inhibitors, show promise but remain under clinical development, underscoring the need for additional effective therapies.

## 2. HDV Is Vaccine-Preventable

Because HDV depends on HBsAg for virion assembly, it cannot establish infection in the absence of HBV. Without concomitant or prior HBV infection, sustained viral spread in humans is not possible. This unique virological interdependence underscores a key preventive implication: effective immunization against HBV concurrently confers protection against HDV infection.

Current HBV vaccines, composed of recombinant HBsAg, elicit robust and long-lasting humoral immunity that prevents HBV infection by neutralizing the surface antigen and blocking viral entry [33]. Consequently, individuals who develop protective anti-HBs antibodies are also protected against HDV infection [34]. This dependency of HDV on HBV positions HBV vaccination as a cornerstone of HDV prevention. While no vaccine exists specifically for HDV, targeting its enabling virus offers a practical and already widely available tool to reduce HDV incidence. A scalable platform for indirect HDV elimination exists through the long-standing global infrastructure for HBV immunization with HBV vaccines via the WHO’s Expanded Programme on Immunization (EPI) [35].

The recommended HBV vaccination schedule includes three doses, which are crucial for establishing strong and lasting immunity. For newborns, many countries implement schedules starting at birth, followed by subsequent doses in infancy; for example, the WHO-recommended schedule is a birth dose followed by two or three doses at 6, 10, and 14 weeks, depending on national guidelines [36]. This is especially relevant in preventing vertical (mother-to-child) and early childhood transmissions, which manage lifelong chronic HBV and later HDV risk.

Epidemiological evidence supports this relationship: in regions with high HBV vaccination coverage, a marked decline in HDV prevalence has been observed, particularly among younger age groups born after the introduction of universal HBV immunization programs [37,38]. Thus, widespread implementation of HBV vaccination not only reduces the global burden of hepatitis B but also serves as a primary preventive strategy against hepatitis D.

## 3. HBV Vaccination Impacts on HDV Prevalence

Real-world epidemiological data from countries with longstanding universal HBV immunization programs provide strong indirect evidence for the impact of HBV vaccination on HDV epidemiology. Widespread HBV vaccination reduces the number of chronic HBV carriers, thereby limiting the population susceptible to HDV co-infection and superinfection. As a result, declines in HBV prevalence in the general population translate into reduced HDV prevalence among HBV carriers, even in the absence of an HDV-specific vaccine.

To clarify this relationship, Table 1 summarizes representative country-level evidence distinguishing (i) reductions in HBV prevalence in the general population from (ii) reductions in HDV prevalence among HBsAg-positive individuals.

The introduction of universal HBV vaccination in Italy in 1991 led to a profound and sustained reduction in HBV transmission. The prevalence of chronic HBsAg carriers in the general population declined from approximately 3% in the 1980s to about 1% by 2010, with a further reduction to 0.33% among children under five years of age by 2020, reflecting the long-term success of infant immunization [39]. This marked decline in HBV prevalence was accompanied by a substantial reduction in HDV prevalence among HBV carriers, which decreased from over 25% in 1983 to approximately 3.2% in recent years [40,41]. The decline in HDV prevalence was particularly pronounced among individuals younger than 30 years, corresponding to cohorts directly protected by the universal vaccination program [52].

Taiwan is one of the first countries to have implemented universal neonatal HBV vaccination in 1984. Taiwan saw the prevalence of HBsAg drop from 10–15% to under 1% over three decades [42]. In Southern Taiwan, HDV superinfection among HBsAg carriers declined from 24.7% in 1990 [43] to 15.3% in 2003 [44], reflecting reduced transmission opportunities as the HBV carrier pool contracted.

In Mongolia, universal infant HBV vaccination was introduced in 1991. Among children, HBV infection rates declined from 31% in 1995 (HBsAg carriage 13.6%) [45] to 15.8% in 2007 (HBsAg carriage 5.2%) [46]. However, despite this substantial reduction in pediatric HBV infection, the downstream effect on HDV prevalence has not yet been formally studied, highlighting an important knowledge gap.

In Iran, national universal childhood HBV vaccination initiated in 1993 reduced HBsAg prevalence from approximately 3.5% pre-vaccination to 0.6% in vaccinated cohorts [47]. Correspondingly, anti-HDV prevalence among asymptomatic HBV carriers declined from 14% in Southern Iran in 1989 [48] to 2.4–9.7% in later regional reviews [49].

Universal neonatal HBV vaccination was introduced in China in 1992 and has been a cornerstone of the national immunization program. National serosurveys show that the prevalence of HBsAg in the general population declined from about 9.7% in 1992 to approximately 5.9% in 2020, with an even steeper drop among young children: HBsAg prevalence in children aged 1–4 years fell from ~9.7% to ~0.30% over this period, reflecting the long-term impact of newborn vaccination and prevention of mother-to-child transmission [50]. These trends demonstrate substantial progress in reducing the HBV reservoir in China, which modeling studies suggest will also contribute to decreased HDV transmission as vaccine coverage remains high [51].

Importantly, despite these successes, HDV prevalence may remain high among older, unvaccinated HBV carriers in several settings. This reflects historical transmission dynamics predating vaccination programs and underscores that HBV immunization primarily prevents new HDV infections rather than eliminating HDV in existing HBV-infected populations.

Together, these data highlight the cascading benefits of HBV immunization not only in preventing HBV transmission but also in indirectly reducing HDV incidence, morbidity, and mortality while emphasizing the need for targeted screening and management of HDV among older HBV carriers.

## 4. Policy Landscape and Existing Gaps

Efforts to control HDV have historically lagged behind those targeting other hepatitis viruses. This delay is largely due to HDV’s dependence on HBV, limited awareness, diagnostic challenges, and the perception that HDV is rare or not cost-effective to address. Consequently, HDV has often been absent from national hepatitis strategies, receiving limited political attention and funding.

### 4.1. Global-Level Gaps

In 2016, the World Health Organization (WHO) launched the Global Health Sector Strategy (GHSS) on Viral Hepatitis, setting the goal of eliminating viral hepatitis as a public health threat by 2030. The strategy aims for a 90% reduction in new chronic infections and a 65% decrease in hepatitis-related mortality compared with 2015 levels [53]. HBV plays a pivotal role in this strategy, with universal vaccination, especially the timely birth dose, identified as one of the most crucial tools for prevention. HDV is not specifically mentioned in the 2016 strategy, and this omission has contributed to limited political visibility, funding and prevention frameworks.

Recent updates have begun to address this gap. The 2024 WHO guidelines mention HDV and the need for screening of all HBsAg carriers [54], and the 2022-2030 GHSS update emphasizes integration of HBV/HDV co-infections into broader hepatitis programs [55]. Despite these advances, implementation is still uneven, and guidance alone has not yet translated into widespread national policy adoption.

### 4.2. Country-Level Gaps

At the national level, HDV-specific policies are extremely limited. A 2022 World Hepatitis Alliance survey found that fewer than 10% of WHO Member States had formal HDV policies, and only 16 countries included HDV in routine hepatitis monitoring systems [56]. Where national hepatitis plans exist, most do not incorporate HDV-specific targets, budgets, or activities.

**Figure 1:**
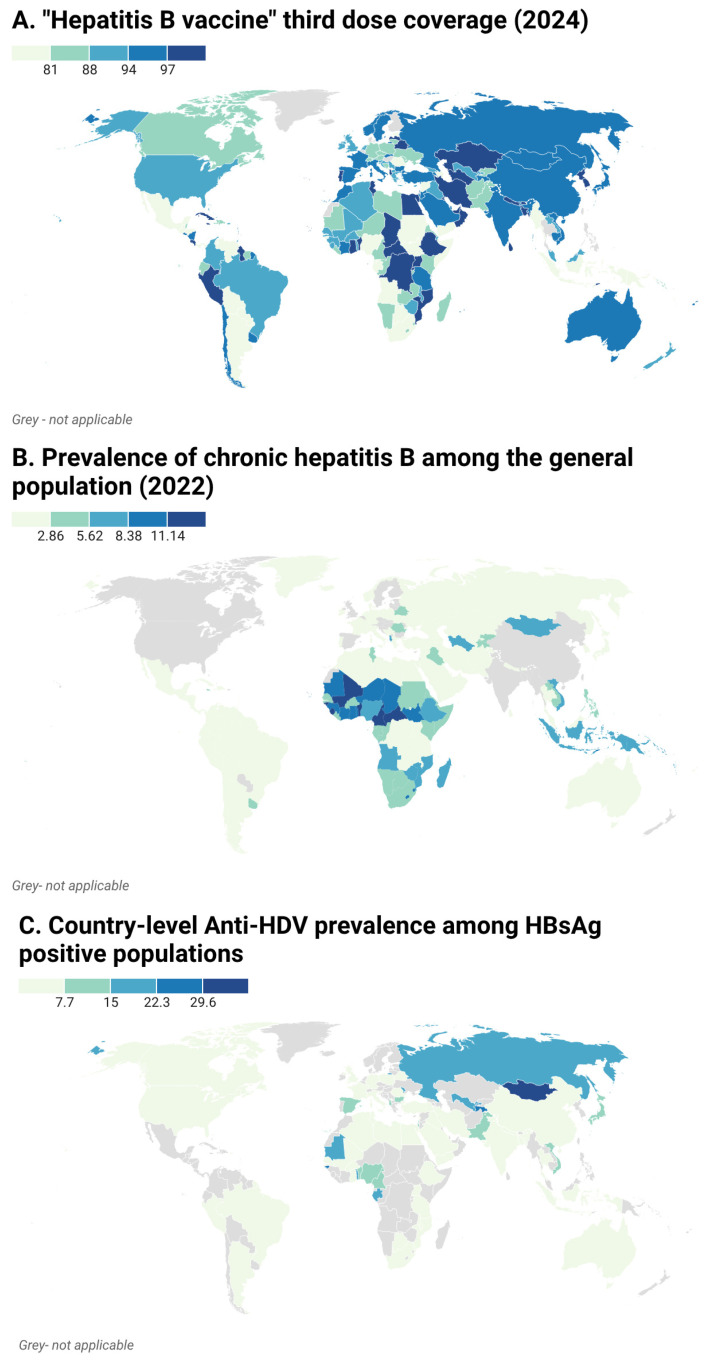
(**A**): Hepatitis B vaccine 3rd dose coverage (the percentage in the target population who have received three doses of Hepatitis B containing vaccine in 2024) reported annually through the WHO/Joint Reporting Form (JRF) on Immunization (2024) [58]. Estimates are based on WHO/United Nations International Children’s Emergency Fund (UNICEF) modelled data and may be affected by data quality variability, reporting lags, and limited subnational or HDV-specific resolution. (**B**): Percentage of chronic hepatitis B among the general population at country-level from World Health Organization Data (2022) [59]. Estimates are based on WHO modelled data and may be affected by variable data quality, modelling assumptions, and limited subnational resolution. (**C**): Anti-HDV prevalence among HBsAg-positive populations at the country-level (review of 2020) [41] Estimates are based on pooled heterogeneous studies, largely relying on anti-HDV serology and limited data from some regions, which may affect comparability and precision. Created with Datawrapper (https://app.datawrapper.de/ accessed on 30 November 2025).

Vaccination policies, while often aligned with WHO recommendations, are not universally enforced or sufficiently funded. Some high-prevalence regions still lack timely HBV birth dose policies, which could dramatically reduce both HBV and HDV transmission [54,57] (Figure 1).

## 5. Challenges

Several hurdles have been hindering the full integration of HDV into national hepatitis policies. Firstly, the absence of population-based HDV prevalence estimates makes it difficult for policymakers to prioritize interventions and to fund research on HDV. Secondly, diagnostic limitations, such as the lack of simple, affordable HDV tests, constrain surveillance and programmatic actions. Therefore, in many countries, HDV is considered a rare or marginal problem, leading to its exclusion from health planning and funding priorities.

### 5.1. Gaps in HDV Surveillance and Diagnosis

One of the most pressing barriers to HDV elimination is the lack of reliable epidemiological data. HDV is not routinely included in viral hepatitis surveillance systems in most countries, making it difficult to accurately measure disease burden, watch trends, or distribute resources. Therefore, the WHO guidelines now recommend universal anti-HDV antibody screening in all HBsAg-positive individuals, followed by HDV RNA in those anti-HDV-positive, and support laboratory reflex testing (anti-HDV immediately after HBsAg, then HDV RNA) where possible [53]. European Association for the Study of the Liver (EASL) clinical-practice guidelines strongly endorse one-time screening of all HBsAg-positive persons for anti-HDV, with repeat testing in patients with ongoing risk or unexplained alanine aminotransferase (ALT) flares, and recommend HDV RNA confirmation using sensitive polymerase chain reaction (PCR) assays [60]. The American Association for Study of Liver Diseases (AASLD) guidance advises screening HBsAg-positive people with risk factors or clinical indications, but does not mandate one-time universal anti-HDV testing for all HBV carriers [61].

Many clinicians do not test HBsAg-positive individuals for HDV due to low awareness or testing protocols. As a result, HDV remains underdiagnosed, and many patients with HDV-related liver disease are misclassified as having HBV monoinfection. A WHO-commissioned survey showed that among health-care workers, only ~20% reported routine anti-HDV testing among HBsAg-positive patients (and even fewer in regions such as sub-Saharan Africa) [61]. Alongside this, in a population of 4385 HBsAg-positive patients in Barcelona, only 8.2% were screened for HDV (anti-HDV antibody) despite guideline recommendations [62].

Testing of the anti-HDV antibody and HDV RNA remain unavailable or expensive in many low-resource settings where the endemicity is high [63,64]. Moreover, a lack of rapid and field-appropriate HDV tests restricts screening efforts in rural and underserved populations. One of the challenges regarding such tests are differences in diagnostic criteria across countries, which complicate data comparisons and burden estimates [37]. HDV exhibits substantial genetic diversity, with at least eight recognized genotypes (HDV-1 to HDV-8) that vary geographically and may differ in antigenicity [4]. This diversity can influence the sensitivity and specificity of serological assays, highlighting the importance of evaluating false-positive and false-negative rates across different genotypes. The development of such genotype-inclusive rapid diagnostic tests will be critical to improve field-based HDV surveillance and exact burden estimation globally.

### 5.2. Vaccine Coverage Issues, Especially in High-Prevalence Regions

While the HBV vaccine is effective and widely available, gaps in vaccination coverage, especially the timely birth dose, leave millions vulnerable to HBV and, by extension, HDV infection. Several high-prevalence regions stay behind on vaccine targets:

In Mongolia, WHO/UNICEF estimates indicate that among children aged 24–35 months, coverage is roughly 91.9% for the first dose and about 87.2% for the third dose [58]. Despite the high vaccination coverage rate, a recent study found that HBV vaccines in rural Mongolia may have been damaged by freezing during winter transport and storage. Children who received all three doses of HBV vaccine in winter had over twice the risk of HBV infection or HBsAg carriage: vaccine-induced immunity was significantly lower among rural children vaccinated in winter (11.3%) compared to non-winter (20.6%), though no difference was seen in urban areas [46]. Another study confirmed HBV vaccines were exposed to freezing temperatures during transport with ice packs from deep freezers, though it did not assess seasonal effects [65].

Vaccine confidence across Europe and Central Asia is relatively low [66], with a 2022 report noting sharp declines, especially in the Baltic states [67]. UNICEF-supported behavioral research shows that parents who trust vaccines’ safety and their healthcare providers are more likely to vaccinate their children on schedule [68].

Many countries in Africa still do not administer the HBV birth dose as part of routine immunization. According to WHO data, only 14% of infants in the African Region received a birth dose in 2022 [69]. In addition, conflict and humanitarian settings, including war zones, refugee camps, and displaced populations often miss routine immunizations due to disrupted health services and loss of records, making HBV and HDV transmission more likely.

### 5.3. Stigma and Awareness

HDV remains unfamiliar to the public, and many health professionals are also unaware of its transmission, clinical severity, or treatment implications. Stigma toward individuals living with HBV and HDV stems from various sources, including prejudices that they may live in unsanitary conditions, use injectable drugs, or have multiple sexual partners. It is also fueled by an unfounded fear of infection, often driven by limited awareness and misunderstanding of how HBV and HDV are transmitted [70]. A recent study performed in Philadelphia, USA showed that stigma surrounding drug use and harm reduction created a substantial obstacle to accessing care [71]. Stigma related to viral hepatitis can further discourage individuals from seeking testing or care [56].

### 5.4. Therapeutic Limitations

Until recently, there were no approved treatments for HDV beyond off-label use of pegylated interferon which yields sustained viral responses in only a minority of patients (15–30%) and is frequently limited by systemic toxicity, reducing its suitability as a population-level strategy [72].

The introduction of BLV represents a major advance. BLV, a large lipopeptide that mimics a 47–amino acid sequence of the HBsAg preS1 region blocks viral entry by competing for binding to NTCP, the receptor used by both HBV and HDV. Authorized in the European Union (EU) [73], BLV demonstrates meaningful on-treatment suppression of HDV RNA [74]. Responses can vary depending on genetic background and immune status [28] and prolonged therapy is often required as viral rebound frequently occurs upon discontinuation [25,26]. Combination of BLV with Peg-IFN has shown higher rates of sustained virologic response compared to BLV monotherapy [31]. However, regulatory approvals are geographically uneven and BLV’s high cost, parenteral route and cold-chain requirements limit access in many low- and middle-income countries (LMICs) [73].

A current phase 2 clinical trial with tobevibart, a monoclonal antibody against HBsAg, and elebsiran, a small interfering RNA that targets HBsAg, showed a decrease in HDV RNA and ALT levels over 48 weeks [32]. Also, nucleic acid polymers such as REP-2139 [75], pegylated-IFN-λ [76] in phase II/III testing, and small NTCP-targeting molecules under preclinical development are offering hope for more tolerable, scalable and possibly oral solutions. But widespread availability will depend on successful late-stage trials, pricing and implementation strategies.

While HBV vaccination remains the cornerstone of HDV prevention, it does not address the existing disease burden in adults already infected with HDV. Therapeutic interventions are therefore essential and should be considered complementary to vaccination in a comprehensive HDV elimination strategy, particularly for high-risk or chronically infected populations.

## 6. Recommendations and Best Practices

### 6.1. Strengthening Birth Dose HBV Vaccine Programs

According to the WHO, timely administration of HBV birth dose (HepB-BD) within 24 h of birth, followed by completion of the vaccine series, can prevent up to 95% of HBV infections in neonates [35]. To strengthen HepB-BD programs, integrating HBV vaccine into newborn care packages and delivery protocols is essential. In China, this has been put into practice by integrating HepB-BD into national immunization and maternal–child health programs, which led to a 97% reduction in chronic HBV infection among children under five [77]. Regarding the delivery of vaccination, training midwives and community birth attendants to vaccinate infants, particularly during out-of-facility births, especially in rural areas, improves the vaccination rate.

Importantly, expanding use of compact prefilled auto-disable injection devices (CPADs) to facilitate delivery in low-infrastructure settings has been leading to efficient vaccination. To mention one example, Vietnam and Senegal introduced CPADs and solar-powered cold chains and increased birth dose coverage in rural regions [78]. Other opportunities to empower vaccine programs is leveraging digital health platforms and birth registries to track vaccination coverage and identify missed doses and community engagement and education to raise awareness of the importance of the birth dose.

Nowadays, non-governmental organizations play a crucial role. For example, the Hepatitis B Foundation [79] and the Coalition for Global Hepatitis Elimination [80] raise community awareness, combat stigma, and support vaccination through regular events and workshops. For instance, the Hepatitis B Foundation has partnered with civil society groups in Nigeria to deliver educational programs to pregnant women and healthcare staff, thereby increasing demand for the hepatitis B birth dose.

### 6.2. Integrated HBV/HDV Elimination Strategies

Integrating HDV within existing HBV and liver disease frameworks is more efficient and sustainable than addressing it separately, such as routine HDV screening for all HBsAg-positive individuals, particularly among high-risk groups such as people who inject drugs, migrants from endemic regions, and individuals with liver disease of unknown etiology [41]. To achieve this integration, cross-training for healthcare providers on HDV pathophysiology, diagnostics, and linkage to care is emerging. A recent study found that the prevalence of anti-HDV among HBsAg-positive patients was notably high in a major Austrian General hospital in Vienna [81]. These findings support the implementation of a double reflex testing strategy, in which anti-HDV testing is automatically performed when HBsAg is detected, and HDV-PCR testing follows if anti-HDV results are positive. In line with 2023 WHO guideline recommendations, European countries such as Germany and Italy have introduced reflex HDV testing protocols, which automatically trigger HDV testing following a positive HBsAg result. This strategy has significantly enhanced detection rates [82].

Although diagnostic assays for HDV are available worldwide, there are substantial gaps: for instance, in a multi-country survey across Africa, anti-HDV testing was reported to be available in only 69% of responding countries, and HDV RNA testing was rarely accessible outside specialized or research laboratories [83]. Moreover, despite existence of HDV RNA quantification assays and a reference standard, heterogeneity in assay design, sample preparation, and genotype variability undermines standardization; indeed, inter-laboratory external quality assessments have demonstrated considerable variability in detection and quantification, particularly for non-genotype-1 strains [84]. At present, no rapid assay for the detection of HDV RNA is available, while such tests exist for several other viral infections. The development of a rapid HDV RNA test would be of considerable clinical value, particularly for guiding patient management and treatment decisions in resource-limited settings [84]. While a rapid point-of-care assay for the serological diagnosis of HDV infection has recently been developed [85], a rapid HDV RNA test would face challenges and performance requirements comparable to those of conventional molecular assays.

### 6.3. Policy Models for Low- and Middle-Income Countries: Mongolia’s National Hepatitis Prevention, Control, and Elimination Program

Mongolia presents a compelling case for LMICs with high HBV and HDV prevalence. Despite limited resources, the country has implemented a comprehensive national hepatitis program emphasizing HBV/HDV prevention, testing, and treatment [86]. This program includes nationwide vaccination campaigns, achieving > 95% infant HBV vaccination coverage, including outreach to nomadic populations. Secondly, population-based screening programs using WHO-recommended testing algorithms for HBV and HCV were performed. According to the Mongolian audit report, 1.1 million people have been screened and around 100,000 anti-HCV-positive and 100,000 HBsAg-positive people have been diagnosed [87]. However, thorough HDV screening remains to be implemented in this country. Thirdly, Mongolia presented an example of public–private partnerships supporting affordable antiviral access and laboratory capacity building. These initiatives demonstrate that strong political commitment, integration across health sectors, and community engagement can yield meaningful progress even in resource-constrained settings.

### 6.4. Global Health Financing and Partnerships

Sustained financing and collaboration are central to HDV elimination. Global health partners such as Gavi, the Vaccine Alliance, the World Bank, and UN agencies play critical roles in expanding access and innovation. For instance, Gavi’s hepatitis B birth dose program in 2024 scaled up funding for birth dose vaccination, particularly in Africa and Central Asia [88].

One of the opportunities to support vaccination is via technology transfer and local vaccine manufacturing to build regional independence. However, several obstacles limit such support. These include difficulties in maintaining funding and barriers to international collaboration. Engaging civil society and patient advocacy groups is essential to reduce stigma and increase demand for hepatitis services. This approach is reinforced by a recent meta-analysis of open-call submissions, primarily from LMICs, which highlighted community-led initiatives in which patients established and managed local organizations to advocate for better access to hepatitis care [89].

The WHO GHSS on Viral Hepatitis (2022–2030) and the World Hepatitis Alliance’s “Find the Missing Millions” campaign provide a global framework to ensure HDV receives appropriate policy attention [55]. Integrating these innovations within strengthened health systems and global partnerships offers a realistic pathway toward eliminating HDV as a public health threat.

## 7. Conclusions

The eradication of HDV relies fundamentally on the successful prevention of HBV infection through robust vaccination programs. The strong biological link between HBV and HDV makes HBV immunization a cornerstone strategy not only to reduce HBV-related morbidity but also to indirectly prevent HDV transmission and disease.

Despite the availability of an effective HBV vaccine and mounting evidence from countries with universal vaccination programs showing dramatic declines in HDV prevalence, particularly in the younger, vaccinated age groups, significant gaps remain. These include insufficient HDV surveillance, diagnostic limitations, uneven vaccine coverage, and the absence of HDV-specific policies within national and global hepatitis elimination frameworks. High-prevalence regions, such as Central Asia and sub-Saharan Africa, particularly suffer from these deficits.

To accelerate HDV elimination, coordinated global and national action is essential. As most screens were based on seroprevalence by antibody testing, more information is needed globally to know the exact prevalence of patients with a currently active infection, i.e., with detectable serum HDV RNA. Strengthened surveillance, including routine HDV testing among HBsAg-positive individuals, will help address data gaps. Timely HBV birth dose vaccination, especially in resource-limited and high-burden settings, is a key prevention strategy. Integrating HDV screening and care within existing HBV and viral hepatitis programs can enhance diagnosis, monitoring, and access to emerging treatments. Accelerating access to currently available HDV treatments, particularly in low- and middle-income countries where the virus is highly endemic, should be a global priority. In countries where treatment is not available, active screening may help to raise awareness to lobby for access to treatment.

National policies that explicitly include HDV in viral hepatitis elimination goals, supported by dedicated funding, awareness campaigns, and capacity building, are critical for sustained progress. Moreover, investment in research and innovation for affordable diagnostics, therapies, and optimized vaccine delivery should be prioritized. Finally, strong multisectoral partnerships among governments, global health agencies, civil society, and affected communities will be vital to maintain momentum toward HDV elimination by 2030.

Key Points:

1. HDV elimination relies fundamentally on HBV prevention via vaccination.
2. Universal HBV immunization reduces both HBV and HDV prevalence, morbidity, and mortality.
3. Key gaps: insufficient surveillance, limited diagnostics, uneven HBV vaccine coverage, lack of HDV-specific policies.
4. Recommendations:
  ◦ Expand timely HBV birth dose vaccination.
  ◦ Integrate HDV screening and care into existing hepatitis programs.
  ◦ Increase access to emerging therapies in endemic regions.
  ◦ Develop HDV-specific policies, funding, and awareness campaigns.
  ◦ Strengthen multisectoral partnerships to accelerate elimination by 2030.

## Figures and Tables

**Table 1:** Impact of universal HBV vaccination programs on HBV prevalence in the general population and HDV prevalence among HBsAg carriers in selected countries.

Country	HBV Vaccination Program	Decline in HBV Prevalence	Decline in HDV Prevalence Among HBV Carriers
Italy	Universal infant/adolescent (1991)	HBsAg carriers in the general population declined from 3% to 0.33% [39]	Anti-HDV prevalence declined from >25% (1983) to ~3.2% [40,41]
Taiwan	Universal neonatal	HBsAg prevalence from 10–15% to <1% [42]	HDV (anti-HDV) superinfection declined from 24.7% (1990) [43] to 15.3% (2003) [44]
Mongolia	Universal infant (1991)	HBsAg carriage in children from 13.6% [45] to 5.2% [46]	Impact on HDV not yet evaluated
Iran	Universal childhood (1993)	HBsAg from ~3.5% to ~0.6% in vaccinated cohorts [47]	Anti-HDV from 14% (1989) [48] to 2.4–9.7% (2008) [49]
China	Expanded HBV coverage	HBsAg carriers in the general population declined from 9.7% to 5.9% [50]	Predicted decline in HDV, especially with >90% birth dose [51]

## Data Availability

No new data were created or analyzed in this study.

## Abbreviations

The following abbreviations are used in this manuscript:

AASLD	American Association for Study of Liver Diseases
CHD	Chronic Hepatitis D
CPADs	Compact prefilled auto-disable injection devices
EASL	European Association for Liver Study
EPI	Expanded Program on Immunization
GHSS	Global Health Sector Strategy
HBV	Hepatitis B Virus
HCC	Hepatocellular carcinoma
HDV	Hepatitis Delta Virus
HepB-BD	HBV birth dose
JRP	Joint Reporting Form on Immunization
LMICs	Low- and Middle-Income Countries
NTCP	Sodium Taurocholate co-transporting polypeptide
UNICEF	United Nations International Children’s Emergency Fund
